# Raman and SERS Spectra of Human Myelin Basic Protein in Cerebrospinal Fluid

**DOI:** 10.3390/nano16100594

**Published:** 2026-05-12

**Authors:** Antonio Bravo-Oro, Sergio Ugarte-Anchondo, Erick Osvaldo Martínez-Ruiz, Ma. del Carmen Rodríguez-Aranda, Adán Reyes-Reyes, Cristian Israel García-Mendoza, Luis Carlos Ortiz-Dosal, Emmanuel Rivera-Pérez, Juan Andrés Reyes-Reyes, Eleazar Samuel Kolosovas-Machuca, Alejandra Ortiz-Dosal

**Affiliations:** 1 Pediatric Neurology Department, Hospital Regional de Alta Especialidad Dr. Ignacio Morones Prieto, Av. Niño Artillero 141, San Luis Potosí 78240, Mexico; antonio.bravo@uaslp.mx (A.B.-O.); sugartea1026@gmail.com (S.U.-A.); 2 Coordinación para la Innovación y Aplicación de la Ciencia y la Tecnología (CIACYT), Universidad Autónoma de San Luis Potosí, Av. Sierra Leona 550, San Luis Potosí 78210, Mexico; erick.martinez@uaslp.mx (E.O.M.-R.); carmen.aranda@uaslp.mx (M.d.C.R.-A.); emmanuel.perez@uaslp.mx (E.R.-P.); a316156@alumnos.uaslp.mx (J.A.R.-R.); 3 Facultad de Ciencias, Universidad Autónoma de San Luis Potosí, Av. Parque Chapultepec 1570, San Luis Potosí 78295, Mexico; adan.reyes@uaslp.mx (A.R.-R.); a337828@alumnos.uaslp.mx (C.I.G.-M.); 4 Maestría en Ciencia e Ingeniería de los Materiales (MCIM-UAZ), Universidad Autónoma de Zacatecas, 801 López Velarde St. 9800, Zacatecas 98160, Mexico; ortiz.dosal.lc@uaz.edu.mx; 5 Facultad de Ciencias UASLP-SECIHTI, Av. Parque Chapultepec 1570, San Luis Potosí 78295, Mexico

**Keywords:** vibrational spectroscopy, spectral analysis, biomarkers, myelin disorders

## Abstract

Raman spectroscopy (RS) provides detailed information on molecular structure but remains challenging for low-scattering proteins in complex media. Myelin basic protein (MBP) is a key structural component of central nervous system myelin and a clinically relevant molecule in demyelinating disorders; however, to the best of our knowledge, its Raman signature in solution has not been reported. In this work, Raman and surface-enhanced Raman spectroscopy (SERS) were employed to characterize purified human myelin basic protein (MBP) in aqueous solution and cerebrospinal fluid (CSF). Quasi-spherical silver nanoparticles were used as SERS elements, yielding enhancement factors of 10^5^ and increasing sensitivity to MBP-associated spectral changes at low concentrations. The MBP spectrum exhibited vibrational modes primarily associated with amide II and amide III bands, as well as aromatic side-chain contributions. Comparative analysis of MBP, CSF, and MBP-spiked CSF samples revealed significant spectral overlap, limiting discrimination based solely on peak positions. To overcome this limitation, spectral correlation and band-intensity-ratio analyses were applied, revealing reproducible trends associated with increasing MBP content. While individual MBP bands are not exclusive, the observed spectral patterns demonstrate the sensitivity of RS and SERS to MBP-induced spectral changes in CSF. These findings should be interpreted as a proof-of-concept in a single-donor CSF matrix.

## 1. Introduction

Raman spectroscopy (RS) is based on the inelastic scattering of monochromatic radiation (laser light). When a sample is irradiated, energy is exchanged between the excitation light and the molecular vibrations of the molecules under investigation. This produces a measurable shift in the wavelength of the incident light, which is reflected in the Raman spectrum. The position and intensity of the Raman bands are determined by the types of atoms (C, O, N, H, S) bonded in the molecule, their bonding patterns (single, double, triple bonds), and the local environment in which they are found (e.g., hydrogen bonds). Therefore, the observed Raman spectra are akin to “molecular fingerprints,” providing information about the covalent structure, conformation, and local environment of the functional groups [1]. In the last decade, RS has been used in the study of various neurodegenerative diseases [2]—for example, in determining spectral differences in the plasma of patients with early-onset and late-onset Alzheimer’s disease and Lewy body dementia [3]; in the analysis of extracellular vesicles from patients with and without Parkinson’s disease [4]; in the characterization of plasma membranes from fibroblasts isolated from patients with Huntington’s disease [5]; and in the spinal cord in an animal model of amyotrophic lateral sclerosis [6]. The technique has proven to be sensitive, rapid, and non-destructive.

Many disorders of the central nervous system result from pathological changes in myelin structure. These can be categorized as dysmyelination, when the myelin is malformed and defective, and as demyelination, when the initially normal myelin is destroyed. Dysmyelinating conditions, also known as leukodystrophies, generally have a strong genetic component, and therefore, neurological abnormalities appear early in life. Demyelinating disorders, such as multiple sclerosis (MS), neuromyelitis optica, and acute disseminated encephalomyelitis, typically present in adults and can be caused by autoimmune processes and infections, with contributing genetic, environmental, and dietary factors. In addition, other conditions, such as genetic leukoencephalopathies and certain metabolic disorders, also exhibit myelination defects, although myelin alterations occur after abnormal neuronal development, neuronal loss, and profound systemic abnormalities. Disorders such as autism and schizophrenia have recently been associated with hypomyelination, revealing new roles for oligodendrocytes and myelin in nervous system development [7].

MBP is an essential structural protein for the tight compaction of the myelin sheath of the central nervous system. This protein exhibits multiple isoforms generated by both alternative splicing and numerous post-translational modifications. The 18.5 kDa MBP isoform, the main component of the myelin sheath, is an intrinsically unstructured protein with a high proportion (approximately 75%) of random coil structures, but with core elements of alpha-helices and beta-sheets [8]. MBP is one of the major myelin sheath autoantigens and is implicated in animal models of autoimmune neurological disorders and in MS, a human demyelinating inflammatory disease characterized by the active degradation of the myelin sheath. Post-translational modifications of MBP may play an essential role in the pathogenesis of MS; for example, arginine deimination occurs at several sites and is associated with increased MS risk. The degree of MBP elimination (or citrullination) correlates with the severity of MS [1].

To the best of our knowledge, no Raman spectrum of human MBP in solution has been reported; only one report of lyophilized murine MBP is available [1]. Since the protein behaves differently in solution, we designed this study to obtain the spectrum of human MBP in aqueous solution and in CSF.

In this context, surface-enhanced Raman spectroscopy (SERS) offers a powerful strategy to overcome the weak Raman scattering of low-abundance biomolecules in complex biological matrices. Metallic nanoparticles, particularly silver nanoparticles, act as plasmonic nanostructures that amplify local electromagnetic fields and enhance Raman signals from molecules located near their surface. Therefore, the use of quasi-spherical AgNPs as SERS-active substrates may improve the sensitivity of Raman-based detection of MBP-associated spectral changes in CSF, supporting the development of nanomaterial-enabled vibrational spectroscopy approaches for biomolecular analysis in neurological biofluids.

## 2. Materials and Methods

We purchased purified Myelin Basic Protein (MBP), containing 14.2 kDa and 18.5 kDa isoforms, as well as their dimers (13.5% SDS-PAGE, non-reducing conditions), from human brain, high purity (≥95% -SDS-PAGE; Immunoblot-), Cat. No. ALX-200-606-M001, Lot. No. M45G211, Enzo, Farmingdale, NY, USA. It was prepared as serial dilutions in deionized water and in cerebrospinal fluid (CSF). CSF with normal analytics (Supplementary Materials Table S1) was obtained from a volunteer donor. Written informed consent was obtained, and “Hospital Regional de Alta Especialidad Dr. Ignacio Morones Prieto” Ethics and Research Committee approval (Protocol Registration number 75-25).

For SERS experiments, quasi-spherical silver nanoparticles (AgNPs) were purchased (Metrohm Silver colloid, Tokyo, Japan, Article #607506300, MW: 107.87 g/mol).

AFM analysis revealed an average height of 6.2 ± 1.4 nm (*n* = 22), while UV-Vis spectroscopy showed an absorbance peak at 423 nm, as shown in Supplementary Materials Figures S1 and S2, respectively. AgNPs were sonicated for 30 min before SERS experiments.

### 2.1. Preparation of Solutions

Serial dilutions of purified human MBP were prepared with deionized water. The initial MBP concentration, C1 = 70 mg/mL, was considered as 100%. The remaining dilutions were labeled as follows: C3 = 0.7 mg/mL, C5 = 0.007 mg/mL, C7 = 70 ng/mL, C9 = 0.7 ng/mL, and C11 = 0.035 ng/mL, corresponding to 1%, 0.01%, 0.0001%, 0.000001%, and 0.00000005% of C1, respectively. For the spike-in Raman experiments, the protein was added to CSF in a 1:4 proportion. For the SERS experiments, the AgNP colloid was added to the MBP + CSF solution in a 1:1 proportion. Further details can be found in the Supplementary Materials.

### 2.2. Characterization by Raman/SERS

Raman and SERS spectra of the described mixtures were acquired by depositing the solutions into the wells of an anodized aluminum plate. An aliquot of 50 µL of each mixture was used. Measurements were performed using a confocal Raman spectrometer (Xplora Plus, Horiba Scientific, Palaiseau, France) equipped with a 532 nm laser (12.5 mW), a 1200 gr/mm grating, and a 20× objective (NA = 0.4). Each spectrum was recorded with 2 accumulations and an acquisition time of 20 s per accumulation.

The confocal pinhole and entrance slit were set to 300 µm and 100 µm, respectively. The spectral resolution was 1 cm^−1^. Instrument calibration was performed using a silicon wafer based on the characteristic band at 520.5 cm^−1^. Spectra were collected over the range of 400–2500 cm^−1^.

### 2.3. Spectral Analysis

After acquiring Raman spectra in the fingerprint range (~400–1800 cm^−1^) for purified MBP, control CSF, and CSF + MBP at different concentrations (C3, C5, C7, C9, C11), a direct comparison of spectra was performed, along with a qualitative analysis of MBP vs. CSF.

Raman spectra were processed and analyzed using the software Fityk (v.1.3.1) [9]. Prior to spectral fitting, all spectra were normalized by the area-under-the-curve method to enable comparison of relative band intensities among samples. Background fluorescence was removed by baseline subtraction using polynomial fitting; the polynomial order was selected individually for each spectrum according to the curvature and magnitude of the fluorescence contribution.

Following baseline correction, the Raman bands were deconvoluted using a combination of pseudo-Voigt and Lorentzian line shapes, providing an accurate representation of the experimentally broadened spectral features.

### 2.4. Evaluation of Spectral Similarity (Correlation) Between Each Sample and Pure MBP

A differential analysis was performed by calculating difference spectra (Cx–control CSF) to isolate contributions attributable to MBP addition. Regions with consistent contrast between MBP and CSF were prioritized, with the analysis primarily focusing on the Amide II and Amide III regions, in accordance with previous Raman studies on MBP and proteins in biological matrices [1]. A panel of SERS spectral intensity ratios (I_1534_/I_1423_, I_1534_/I_1312_, I_1584_/I_1442_) was defined to minimize background effects and instrumental variations, following strategies reported in clinical Raman studies.

## 3. Results

**Figure 1:**
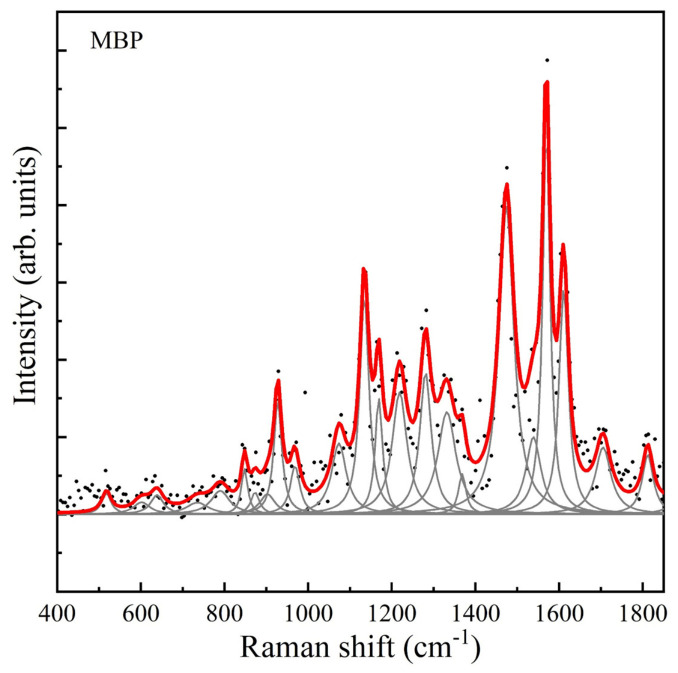
Raman spectrum of human myelin basic protein (MBP) in aqueous solution, at a concentration of 70 mg/mL. Black dots correspond to the baseline-free spectrum, gray curves correspond to the found pseudo-Voigt and Lorentzian line shapes, and the red curve corresponds to the final fitted spectrum.

**Figure 2:**
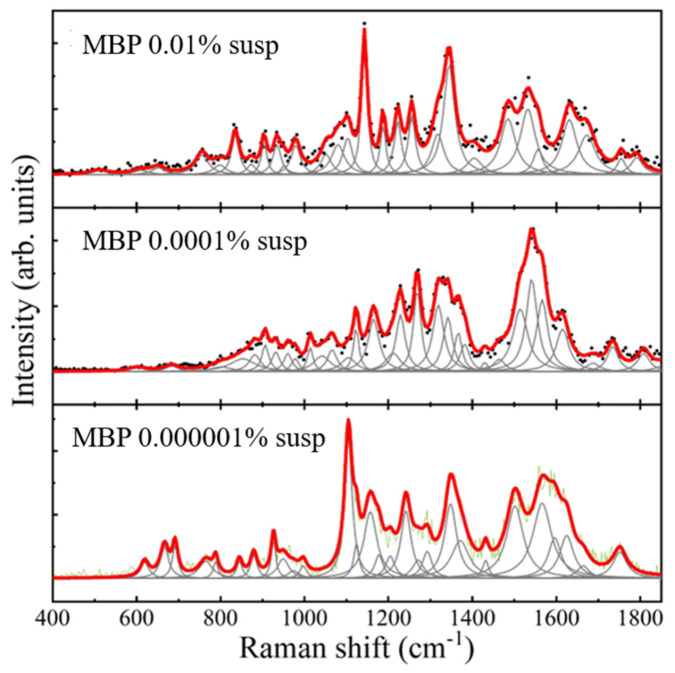
Raman spectrum of myelin basic protein in aqueous solution, at decreasing concentrations: 0.007 mg/mL (0.01%, **top panel**), 0.00007 mg/mL (70 ng/mL, 0.0001%, **middle panel**), and 0.000007 mg/mL (0.7 ng/mL, 0.000001%, **bottom panel**). In each of these panels, black dots/green curves correspond to the baseline-free spectrum, gray curves correspond to the found pseudo-Voigt and Lorentzian line shapes, and the red curve corresponds to the final fitted spectrum.

**Figure 3:**
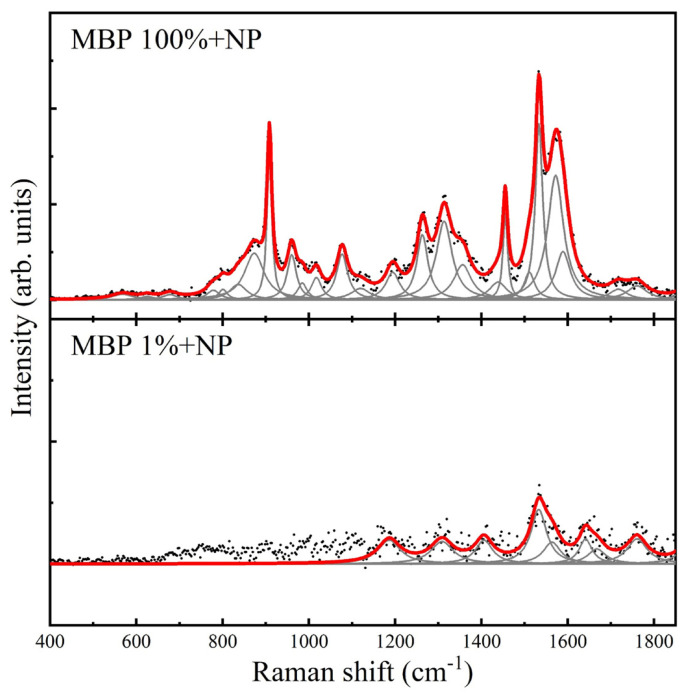
SERS spectrum of myelin basic protein in aqueous solution at a concentration of 70 mg/mL (100%, **top panel**), and 0.7 mg/mL (1%, **bottom panel**). In each of these panels, black dots correspond to the baseline-free spectrum, gray curves correspond to the found pseudo-Voigt and Lorentzian line shapes, and the red curve corresponds to the final fitted spectrum.

The Raman spectra of both MBP in aqueous solution and in CSF were very weak, so silver nanoparticles were added as a reactant to amplify the signal. Following this, the SERS spectrum was clearly identifiable in all measurements, yielding an adequate amplification factor. The maximum enhancement factor was calculated to be 2.69 × 10^5^ (Figure S3, Supplementary Materials). The SERS spectrum of the purified MBP was obtained, with the highest-intensity band at 1572 cm^−1^, as well as lower-intensity bands at 1132, 1475, 1609 cm^−1^, and also at 928, 1073, 1170, 1218, and 1282 cm^−1^ (Figure 1, Figure 2 and Figure 3). The complete relationship between the bands found in the Raman/SERS spectrum of MBP and the proposed vibrational mode assignments is described in Table 1. The band at 1813 cm^−1^ is not typically observed in biological molecules, as it falls in a “silent region” of the Raman spectrum, which is attributed to triple bonds that are uncommon in this type of sample. Vibrations of the bonds of “tags” artificially added during the protein purification process, which may contain an alkyne or nitrile group, may appear in this region [10].

**Figure 4:**
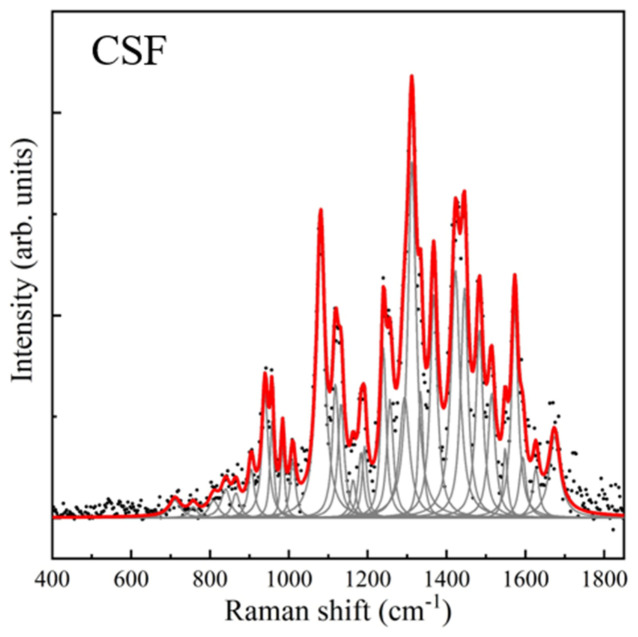
Raman spectrum of cerebrospinal fluid. Black dots correspond to the baseline-free spectrum, gray curves correspond to the found pseudo-Voigt and Lorentzian line shapes, and the red curve corresponds to the final fitted spectrum.

Analysis of the SERS spectrum of cerebrospinal fluid (CSF) revealed the highest-intensity bands in the 1200–1450 cm^−1^ region, with the most intense band at 1312 cm^−1^. Some lower-intensity bands were also identified in the 900–1050 cm^−1^ region (Table 2, Figure 4). This spectrum differs from that reported by other authors [12], likely due to the improved spectral characteristics resulting from the addition of silver nanoparticles.

**Figure 5:**
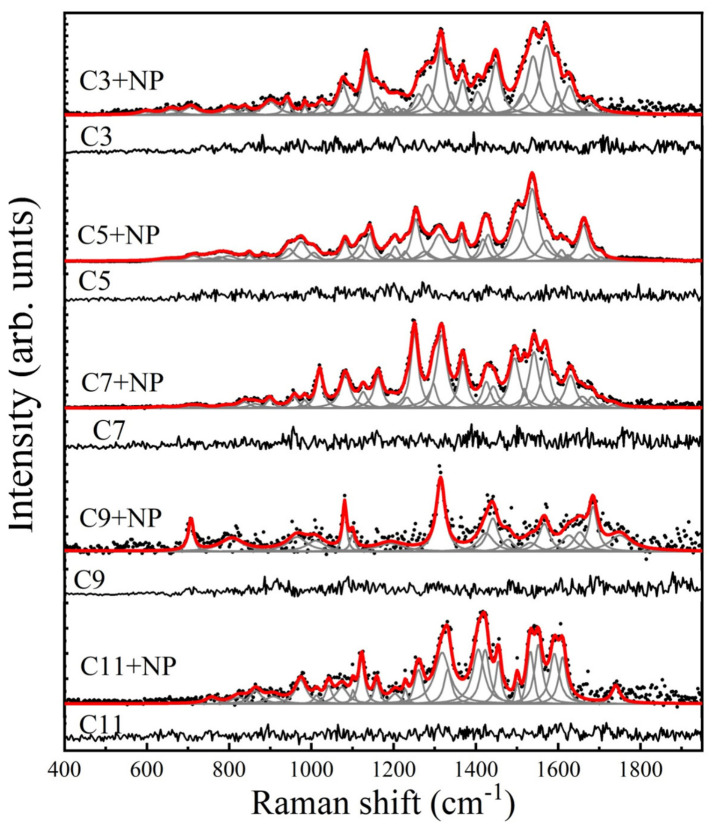
Spike-in experiments. Raman (black) and SERS (red) spectra of myelin basic protein at decreasing concentrations in cerebrospinal fluid. Concentrations C3, C5, C7, C9, and C11 correspond to 0.7 mg/mL, 0.007 mg/mL, 70 ng/mL, 0.7 ng/mL, and 0.035 ng/mL, respectively. In each of these panels, black dots correspond to the baseline-free SERS spectrum, and the gray curves correspond to the found pseudo-Voigt and Lorentzian line shapes.

When determining the SERS spectra using the spike-in technique, low-intensity bands were observed in the 700–985 region. Interestingly, the band at 708 was more intense at lower protein concentrations (C9 and C11), as reported for other proteins [13]. Higher-intensity bands were found in the 1000–1631 region. The band analysis and proposed band assignment are described in Table 3 and Figure 5.

As a result of the SERS experiments, 19 modes were identified in the MBP spectrum, 27 modes in the CSF spectrum, and 18 modes in the spectra corresponding to the spike-in experiments (Table 4). All the modes obtained in the latter experiment can be related to the modes obtained in CSF without the addition of MBP. However, some bands are common to all three solutions, mainly in the 1070–1170 region, where three vibrational modes coincide in all three solutions analyzed. These modes correspond to C-C and C-N protein constrictions (see Table 1, Table 2 and Table 3). The bands 966/956/959, 1368/1367/1367, 1475/1484/1495, and 1572/1573/1567 also coincide across all three solutions in the MBP, CSF, and spike-in spectra (Table 4). Due to these results, it is not possible to propose an isolated analysis of band position to discriminate the presence of MBP in CSF. However, differences in band intensity and width do exist. Therefore, a spectral ratio analysis, similar to the strategy reported by Sathyavathi et al. (2013) [14], was performed to determine whether a reproducible spectral pattern is associated with MBP in the CSF matrix.

### 3.1. Spectral Ratio Analysis: Comparison of MBP vs. CSF

The pure MBP spectrum shows clear increases in the Amide II (~1534–1562 cm^−1^) and Amide III (~1260–1310 cm^−1^) regions compared to CSF.

CSF shows a greater relative contribution in bands associated with lipid mixtures, carbohydrates, and biological background, as reported in previous Raman studies of CSF.

### 3.2. Spectral Similarity

Samples C3, C5, C7, and C11 show greater similarity to MBP than the control CSF.

There is a consistent trend: samples with higher MBP show a greater correlation with the pure MBP spectrum Sample C9 deviated from the expected concentration-dependent trend and behaved spectrally more similarly to control CSF than to the other MBP-spiked samples. This deviation may plausibly reflect experimental variability, AgNP aggregation effects, matrix interactions, measurements near the detection limit, or possible sample preparation/handling errors. Therefore, C9 was interpreted with caution and treated as a potential outlier. Additional replicated measurements will be required to determine the origin of this deviation and to confirm the concentration-dependent spectral trend.

**Figure 6:**
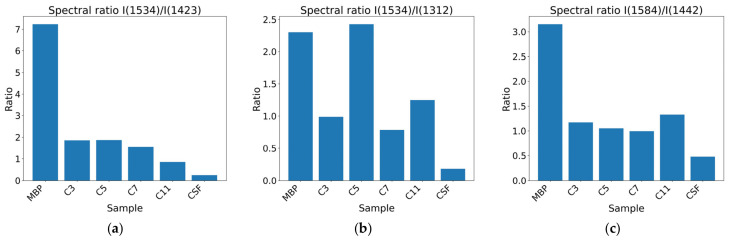
Spectral ratios (**a**) I(1534)/I(1423), (**b**) I(1534)/I(1312) and (**c**) I(1584)/I(1442). The analysis separates the spectrum of purified MBP, control CSF, and the spike-in dilutions.

SERS intensity ratios such as I_(1534)_/I_(1423)_, I_(1534)_/I_(1312)_, and I_(1584)_/I_(1442)_ clearly separate pure MBP, control CSF, and intermediate samples from the spike-in experiments (Figure 6).

They place C3/C5/C7/C11 between MBP and CSF, as expected for mixtures.

When C9 is treated as a potential outlier, the remaining samples exhibit consistent and reproducible behavior, in line with methodologies reported in clinical Raman studies [14].

The band ratio I_(1534)_/I_(1423)_ correlates with CSF MBP concentration, mainly when it is below 0.7 mg/mL.

## 4. Discussion

RS could be a valuable technique for observing MBP’s conformation in solution and for studying its interactions with other molecules and the changes associated with modifications to its secondary structure. This requires analysis of the protein spectrum. In this project, the Raman spectrum of purified MBP was initially obtained in aqueous solution, and the SERS spectrum was subsequently obtained upon addition of a colloidal AgNP suspension. The resulting MBP spectrum allowed for the identification of bands, particularly in the amide III region, which is sensitive to molecular vibrations of the protein’s carbon backbone, as well as to the side chains of the constituent amino acids (Table 1, Figure 1, Figure 2 and Figure 3).

Previous studies have used various RS techniques to evaluate neurological diseases and analyze MBP. In a relatively recent study, Lariosa-Willingham et al. (2022) [7] used an enhanced Raman resonance (RRS) ELISA platform to evaluate a neonatal myelination model in rats. Treatment of rat pups with T4 significantly increased MBP levels compared with vehicle-treated groups after 6 to 9 days of administration. The authors administered thyroid hormone (T4) for 6–9 days and evaluated its promyelinating effect by quantifying MBP in rat pup brain lysates. The ELISA + RRS platform proved helpful, yielding consistent, reproducible results for identifying and quantifying MBP in brain tissue. This is particularly challenging because, while ELISA offers advantages over conventional techniques (Western blot and immunohistochemistry) in terms of speed, throughput, and sensitivity, it also has limitations. Brain tissue is complex and contains “sticky” components that bind nonspecifically to MBP, leading to a high background signal in traditional sandwich ELISA assays. MBP in dilute solutions binds to glass, plastic, specific brain proteins, and red blood cells.

Furthermore, conventional ELISA methods lack sufficient sensitivity to detect the low levels of MBP in the developing rat brain. Therefore, the authors developed an ELISA assay to quantify MBP using a direct detection method with RRS reagents. This new method overcame the problem of nonspecific binding and increased detection sensitivity. The RRS-ELISA demonstrated higher sensitivity than the standard detection approach and more reproducible MBP measurements. The level of MBP in the rat brain was assessed using the RRS-ELISA assay based on the absolute quantification of MBP with a purified MBP standard curve. This study did not aim to characterize rat MBP using RS, but rather to quantify the protein as a method for evaluating pharmacologically induced myelination.

Myelin damage can lead to loss of axonal conduction and paralysis in multiple sclerosis and spinal cord injury. In an animal model of pharmacologically induced demyelination, Shi et al. (2011) [15] demonstrated that acrolein, a lipid peroxidation product, can cause significant myelin damage in isolated segments of the guinea pig spinal cord. This damage was assessed by detecting guinea pig myelin sheath membranes (MBP) using immunofluorescence. A specialized technique, coherent anti-Stokes Raman scattering (CARS), was used to evaluate the myelin sheath at the nodes of Ranvier and in the paranodal regions. This labeling enabled visualization of acrolein-induced myelin damage. However, this technique was not used to describe the MBP spectrum in this study.

de Almeida Melo Maciel Mangueira et al. (2022) [16] determined the Raman spectrum of the sciatic nerve in rats using an animal model of compression injury (axonotmesis-type lesion). In this study, the Raman bands identified as corresponding to the rat sciatic nerve did not match those found for the MBP, except for the 1065 cm^−1^ band (1073 cm^−1^ in our study), attributed to phospholipids, and the 1270 cm^−1^ band (1282 cm^−1^ in our spectrum), attributed to the amide III region, alpha helices, CH2 flutter in the glycine backbone (1279 cm^−1^), and proline side chains (1280 cm^−1^).

In another study, Wang et al. (2011) [1] used recombinant murine analogs of two isoforms of the classical 18.5 kDa MBP as model proteins to understand the structure and function of charge isomers. This characterization included biochemical and biophysical methods, such as size exclusion chromatography, calorimetry, surface plasmon resonance, small-angle X-ray and neutron scattering, Raman and fluorescence spectroscopy, and conventional and synchrotron circular dichroism, to investigate the differences between these two isoforms, both structurally and in terms of their interactions with ligands such as calmodulin, various detergents, nucleotide analogs, and lipids. The results provided critical additional insights into the interactions between MBP and its ligands, as well as the structural and functional differences between its charge isomers.

In the work by Wang et al. (2011) [1], the conformation of lyophilized murine recombinant MBP was analyzed using RS. In contrast, in the present project we analyzed purified human recombinant MBP in solutions, both in deionized water and in cerebrospinal fluid. The Raman spectra in both studies were excited with a 532 nm laser. The structural analysis comparing the results of that study (which, after the systematic review described, is the only one that reports the Raman spectrum of murine recombinant MBP) with those of the present project is discussed below.

### 4.1. Structural Details of the Raman Spectrum of Recombinant Murine MBP and Comparison with Human MBP Results from the Present Study

Aromatic rings are good Raman scatterers, and therefore, the Raman bands characteristic of aromatic amino acids are quite strong and can be identified in the protein spectrum. However, they may overlap with amide bands and other side-chain groups. In the spectrum reported by Wang et al. (2011) [1] of recombinant murine MBP, the phenylalanine ring mode at 1004 cm^−1^ is very characteristic (this band was not observed in the spectrum of human MBP in solution). Other vibrations indicative of phenylalanine in murine MBP were found at 1606 cm^−1^, 1204 cm^−1^, 819 cm^−1^, and 622 cm^−1^. In human MBP, bands indicative of phenylalanine were found at 1609 cm^−1^, 1218 cm^−1^, 848 cm^−1^, and 639 cm^−1^. The band number is practically the same, considering that, in solution, the location of the same vibrational mode can shift up to 33 cm^−1^; in this case, all shifts occurred in the infrared. In murine MBP, vibrational bands assigned to tryptophan were found at 1617 cm^−1^, 1576 cm^−1^, and 1421 cm^−1^, and the Fermi doublet at 1357 cm^−1^ and 1339 cm^−1^, respectively. In the human MBP spectrum, we found bands corresponding to tryptophan bonds at 789 cm^−1^, 875 cm^−1^, 1218 cm^−1^, and 1331 cm^−1^, and the Fermi doublet at 1368 and 1475 cm^−1^. In the case of human MBP, we observed greater intensity in the amide II bands, particularly at 1475 cm^−1^, 1572 cm^−1^, and 1609 cm^−1^ (Figure 1). The assignment of these bands corresponds to molecular vibrations of the C=N and C=C bonds of amino acids with ring structures, specifically tyrosine and phenylalanine (Table 1).

Tyrosine exhibits a Fermi doublet around 850 cm^−1^ and 830 cm^−1^. The intensity ratio of the two bands can be correlated with the hydrogen bonding states of the tyrosine hydroxyl group. The higher the relative ratio (I854/I838 in murine MBP, and I875/I848 in human MBP), the lower the molecule’s capacity to act as a hydrogen bond donor. In murine MBP, more tyrosine Raman bands are found at 644 cm^−1^, 1182 cm^−1^, and 1617 cm^−1^; the latter overlaps with the tryptophan band. In the case of human MBP, we find bands assigned to tyrosine at 639 cm^−1^, 1170 cm^−1^, 1218 cm^−1^ (this one overlapping with phenylalanine and tryptophan vibrations), 1368 cm^−1^ (overlapping with tryptophan), and 1609 cm^−1^ (overlapping with phenylalanine).

MBP exhibits a disordered structure in aqueous solution [8] and tends to fold into a somewhat helical structure after slow drying on a surface. The amide I band is located around 1666 cm^−1^. This vibrational band is due to the C=O stretching mode of the peptide bond. Depending on the secondary structure, the exact wavenumber can range from 1640 to 1680 cm^−1^. If hydrogen bonds form between the C=O and NH groups of different peptide backbones, the amide I band is observed around 1670 cm^−1^. The position of the amide I band also suggests the presence of secondary structures, which could be induced by lyophilization [1]. This phenomenon was observed as a change in the position of the bands in the amide I region of the protein’s Raman spectra in aqueous solution. The human MBP spectrum was obtained in solution, both in deionized water and in CSF. Therefore, different positions were observed in the amide I, II, and III bands, some of which were associated with other secondary structures (alpha helices) than those observed in the spectrum of lyophilized murine MBP. These differences could be attributed to the behavior of the protein in solution, rather than to species differences.

### 4.2. SERS Intensity Ratio Analysis

In complex biological matrices such as CSF, unlabeled RS cannot directly identify individual proteins. Structural studies of proteins using RS demonstrate that amide bands reflect protein content and conformation, but do not constitute exclusive molecular signatures.

The specialized literature on RS applied to biofluids and liquid biopsies indicates that the methodological approach is to analyze spectral patterns, use band ratios, compare against biological controls, and avoid absolute molecular attributions without specific capture or selective spectral radiation. This approach has been successfully applied in clinical CSF studies to detect pathological conditions using Raman signatures, without the need to directly identify individual biomarkers [12].

Therefore, we focused the analysis on detecting a pattern consistent with MBP presence rather than assigning exclusive peaks. Band-level assignments were interpreted within a pattern-based framework. The results of the SERS spectral ratio analysis are consistent with a Raman signature associated with increased protein content compatible with MBP in CSF. The observed bands are not exclusive to MBP. Still, their systematic variation with added concentration supports the method’s sensitivity to MBP-induced changes and supports the use of RS as an exploratory tool. This behavior is consistent with previous reports where RS in CSF detects biochemical patterns associated with biomarkers but does not identify individual proteins without specific amplification or capture strategies. With this experimental strategy, we observed that the I_(1534)_/I_(1423)_ band ratio is directly related to MBP concentration in CSF when it is below 0.7 mg/mL (Figure 6); however, more measurements are needed to confirm this trend.

### 4.3. Applications in Demyelinating Neurological Diseases

A recent study (Neupokoeva et al., 2025) [17] compared the spectral signatures of serum from patients with multiple sclerosis and healthy controls. The authors found differences in the intensities of the 623, 721–735, and 1048–1076 cm^−1^ bands, with discrimination percentages of 60–65% for each band. In this study, the 623 cm^−1^ band was associated with C-H vibrations in phospholipids and cholesterol, components of myelin sheaths. The 721–735 cm^−1^ range was attributed to vibrations in amide bonds, which, according to the study authors, could reflect changes in the peptide structure of proteins such as MBP. The 1048–1076 cm^−1^ region has been attributed to vibrations in the structure of cell membranes and glycoproteins involved in the inflammatory and immune activation processes observed in patients with multiple sclerosis [17].

Contrary to these band assignments, we found no significant bands in the 721–735 cm^−1^ region when analyzing the MBP spectrum. Still, we did see them at 639 cm^−1^ (attributed to C-S vibrations of the amino acids methionine and tyrosine, and C-C vibrations of tyrosine and phenylalanine) and at 1073 cm^−1^ (attributed to vibrations in proline bonds). Therefore, the authors’ consideration of mode attribution in the 721–735 cm^−1^ region may not accurately reflect the MBP structure, and further experimental investigation is necessary to validate this interpretation.

### 4.4. Limitations

Our study has several limitations. It is an initial experimental approach; therefore, it is not possible to determine with certainty whether the results will apply to real clinical situations. The use of spontaneous Raman does not allow for absolute molecular specificity in CSF. Only one cerebrospinal fluid (CSF) sample from a patient with normal laboratory results was used. Therefore, this study should be considered a proof of concept, as there is a lack of statistical replication and quantitative validation, and the analysis of other molecules present in CSF was not considered. The spectral characteristics of individuals with pathological conditions are likely variable; therefore, even if the MBP concentration in these conditions is the same as that evaluated in this project, the protein’s interaction with the different biofluids could give rise to different vibrational modes. Studies with different experimental strategies are required to propose RS as a screening or diagnostic tool in the future.

## 5. Conclusions

Currently, there is a trend toward using not only early diagnostic markers but also indicators found in biological fluids. To the best of our knowledge, no reports indexed in PubMed describe the Raman structure of human MBP in solution. Therefore, this project analyzed Raman spectra of cerebrospinal fluid (CSF) with different concentrations of the MBP. Signal amplification of purified human MBP was achieved using quasi-spherical silver nanoparticles, yielding enhancement factors of approximately 10^5^. The MBP SERS spectra displayed prominent bands in the amide II and amide III regions, along with contributions from aromatic amino acid residues. Adequate signals were also obtained from both the CSF spectrum and the spike-in arrays. Due to substantial spectral overlap with CSF components, MBP could not be identified by analyzing only individual band positions. Instead, spectral similarity metrics and intensity ratios enabled pattern-based discrimination between MBP-spiked samples and control CSF, supporting sensitivity to MBP-induced spectral changes. Given that only one CSF sample was analyzed, these findings should be interpreted as proof-of-concept for exploratory RS-based studies of myelin-related pathology.

## Figures and Tables

**Table 1:** Characteristic Raman spectrum bands of Myelin Basic Protein at a protein concentration of 0.07 mg/mL in deionized water [11].

Raman Shift (cm^−1^)	Proposed Band Assignment
516	rocking of CO_2_^−^ (536) (isoleucine)
639	v(C-S) gauche (methionine) (630–670) C-C twisting of tyrosine and phenylalanine (643–649)
789	Ring breathing tryptophan (proteins) (760) C-C stretching (813)
848	Single-bond stretching vibrations for valine (850) Tyrosine (Fermi resonance of ring fundamental and overtone) Ring breathing mode of tyrosine & C-C stretch of proline ring (853)
875	C-C stretching, hydroxyproline (876) Tryptophan, d(ring) (880)
928	Skeletal C-C, α -helix (932) C-C stretching mode Proline, hydroxyproline, valine (928–950)
966	C-C backbone (971–973) C-C stretching β-sheet (proteins) (980)
1073	Proline (1066–1067)
1132	C-N stretching (1128)
1170	C-H in-plane bending mode of tyrosine (1170)
1218	Amide III C-N stretching, C=N=C stretching, and N-H bending (1200–1350), (β-sheet structure) (1220) Hydroxyproline, tyrosine (1206) v(C-C_6_H_5_), tryptophan, phenylalanine (1208)
1282	Amide III (α-helix) (1279) CH_2_ wagging vibrations from glycine backbone & proline side chains (1280)
1331	CH_3_CH_2_ twisting, wagging, and bending modes (1335–1339) Amide III & CH_2_ wagging vibrations from glycine backbone & proline side chain (1337) Tryptophan (1337–1339)
1368	Tryptophan (1365)
1475	Fermi interaction d(CH2), & g(CH2) (1463) C=N stretching (1470)
1572	Amide II (CN stretching & in-plane bending of the N-H group (1480–1575) C-C stretching (1580) d(C=C), phenylalanine (1582–1583)
1609	Amide I, C=O stretching vibrations of the peptide backbone (1600–1800) C=C in-plane bending mode of phenylalanine & tyrosine (1603–1607)
1706	v(C=O)OH (amino acids aspartic & glutamic acid) (1700–1750)
1813	Silent region Stretching carbonil group C=O (carbonil region 1600–1900)

**Table 2:** Band assignment in the cerebrospinal fluid spectrum [11].

Raman Shift (cm^−1^)	Proposed Band Assignment
711	v(C-S) trans (amino acid methionine) (700–745) Cholesterol, cholesterol ester (702) C-N membrane phospholipids head (717–719), sphingomyelin (719) Symmetrical stretch vibration of the choline group N^+^(CH_3_)_3_ (719) C-C-N^+^ symmetric stretching in phosphatidylcholine (719)
756	Symmetric breathing of tryptophan (752–759)
811	O-P-O stretching RNA (811) C-C stretching proline (815)
839	Asymmetric O-P-O stretching (831) Deformative vibrations of amine groups (838) Polysaccharide structure (840–860)
864	Tyrosine, collagen (859) Phosphate group, phosphatidic acid (860) Ribose vibration, one of the distinct RNA modes (867, with 915 and 974) Carbohydrates (C-O-C) skeletal mode (868) Single bond stretching in proline, valine (869) C-C stretching (868–870)
906	Carbohydrates (C-O-C) skeletal mode (898–913) Proline (918)
939	C-C stretch backbone (938) Skeletal modes (polysaccharides) (941)
956	Single bond stretching in proline, valine, and polysaccharides (950) v_s_(CH_3_) of proteins (a-helix) (951) Lipids (968)
984	C-C stretching b-sheet (proteins) (980) =CH bending (lipids) (980)
1009	Bound & free NADH (1000) Symmetric ring breathing mode of phenylalanine (1000–1034)
1081	Symmetric PO_2_^−^ stretching of DNA (1070–1090) Phosphodiester vibrations in nucleic acids (1080–84) C-C or C-O stretching mode of phospholipids (1078–80) v_1_CO_3_^2−^, v_3_PO_4_^3−^, v(C-C) skeletal of acyl backbone in lipid (gauche conformation) (1081–1087) Carbohydrates (1082) C-N stretching mode of proteins (1083)
1120	Glucose (1117) C-O band of ribose (marker band of RNA in solutions) (1120) C-C stretching mode of lipids and protein, C-N stretch (1117–1130)
1164	C-C/C-N stretching (proteins) (1155–1158) C-H in-plane bending mode of tyrosine (1163–1172)
1189	Cytosine, guanine, adenine (1180–1184) Anti-symmetric phosphate vibrations (1185–1300)
1239	Amide III (1200–1300) (due to coupling of C-N stretching & N-H bonding); ß-sheet and random coils) (1242) CH_2_ wagging vibrations from glycine backbone & proline side chains (1237–1239) Asymmetric phosphate [PO_2_^−^ (asym.)] stretching modes (1243)
1256	C-N in-plane stretching (1254) Lipids (1255) A, T (ring breathing modes of the DNA/RNA bases); amide III (protein) 1257–59
1312	C-N asymmetric stretching in asymmetric aromatic amines (1308) CH_3_/CH_2_ twisting, wagging &/or bending mode of proteins & lipids (1300–14) G (ring breathing modes of the DNA/RNA bases)-C-H deformation (protein) (1318) Amide III (a-helix) (1318–21)
1334	Amide III & CH_3_CH_2_ wagging vibrations from glycine backbone & proline side chain (1335–45) A, G (ring breathing modes in the DNA bases)- C-H deformation (protein) (1337) Tryptophan (1337–9)
1367	Tryptophan (1359–69) v_s_(CH_3_) (phospholipids) (1367)
1423	v(C=O)O^−^ (amino acids aspartic & glutamic acid) (1400–30) CH_2_ bending and scissoring modes of proteins & lipids, CH deformation (DNA/RNA & proteins & lipids & carbohydrates) (1420–80) NH in-plane deformation (1423)
1445	CH_2_ deformation (1437–53), scissoring (1439) in proteins and lipids
1484	Amide II (due to a coupling of CN stretching & in-plane bending of the N-H group) (1480–575) G, A (ring breathing modes in the purine DNA bases) (1485)
1515	Cytosine (1515) In-plane vibrations of the conjugated -C=C- (1525)
1548	v(C=C), tryptophan (1548–1560) v(CN) and d(NH) amide II (protein assignment) (1558) Tyrosine, COO^−^ (1558)
1573	G, A (ring breathing modes of the DNA/RNA bases) (1573–75) d(C=C), C=C bending mode of phenylalanine (1582–83)
1626	Amide I band of proteins, due to C=O stretching (1600–800) Tryptophan and/or ß-sheet (1622) C¤=C¤ stretch (1628) Amide C=O stretching absorption for the ß -form polypeptide films (1628)
1673	C=C stretch (1670–4) Amide I band (C=O stretch coupled to a N-H bending) (1673) Amide I (ß -sheet) (1676)

**Table 3:** Bands obtained in the SERS spike-in experiment C7 to C11 + CSF (MBP concentration in the solution: 7 ng/mL to 0.00007 ng/mL) [11].

Raman Shift (cm^−1^)	Proposed Band Assignment
708	v(C-S) trans (aminoacid methionine) (700–745) Cholesterol, cholesterol ester (702) CN_2_ (CH_3_)_3_ (lipids) (717–719) Choline group (717–719) C-N (membrane phospholipid head)/nucleotide Peak (718) Symmetrical stretch vibration of the choline group N^+^(CH_3_)_3_, characteristic of phospholipids (719) Phosphatidylcholine, sphingomyelin (719) C-C-N^+^ symmetric stretching in phosphatidylcholine (lipid assignment) (719)
858	Tyrosine (Fermi resonance of ring fundamental and overtone) (850) C-C stretch of proline ring, hydroxyproline, tyrosine ring breathing mode (852–856) Glycogen (853) Amino acid side chain vibrations of proline & hydroxyproline, as well as a (C-C) vibration of the protein backbone (856) Tyrosine (859) Phosphate group (860) Phosphatidic acid (860) Ribose vibration, one of the distinct RNA modes (867, with 915 and 974) C-C stretching (868) Monosaccharides (b-fructose), (C-O-C) skeletal mode (868) Disaccharide (sucrose), (C-O-C) skeletal mode (868) Polysaccharides, amylose (868) Polysaccharides, amylopectin (868) Proline (869) Most probably due to single-bond stretching vibrations for the amino acids proline and valine and polysaccharides (870) C-C stretching in proteins (870)
897	Hydroxyproline, tryptophan (879) Tryptophan, d(ring) (880) Saccharide band (overlaps with acyl band) (891) Backbone, C-C skeletal (893) Phosphodiester, Deoxyribose (893–896) Monosaccharides (β-glucose), (C-O-C) skeletal mode (898) Disaccharide (maltose), (C-O-C) skeletal mode (898)
959	Most probably due to single-bond stretching vibrations for the amino acids proline and valine, and polysaccharides (950) v_s_(CH_3_) of proteins (a-helix) (951) Lipids (968)
984	C-C stretching b-sheet (proteins) (980) =CH bending (lipids) (980)
1020	v(CO), v(CC), d(OCH), ring (polysaccharides) Symmetric ring breathing mode of phenylalanine (1000–1034) Glycogen (1022–1025)
1084	Symmetric PO^2−^ stretching of DNA (represents more DNA in the cell) (1070–1090) v(C-C) or v(C-O), phospholipids (lipid assignment) (1078) Pronounced symmetric phosphate stretch (1078) Phospholipids (1078) C-C or C-O stretching mode of phospholipids (1078) Carbohydrate peak for solids (1078) C-C or C-O stretch (lipid), C-C or PO2 stretch (nucleic acid) (1078) Typical phospholipids (1080) Phosphate vibrations (phosphodiester groups in nucleic acids) (1080) v_1_CO_3_^2−^, v_3_PO_4_^3−^ v(C-C) skeletal of acyl backbone in lipid (gauche conformation) (1081) Carbohydrate residues of collagen (1082) Carbohydrates peak for solutions (1082) Nucleic acids (1082) C-N stretching mode of proteins (and lipid mode of lesser degree) (1083) Phosphodiester groups in nucleic acids (1084) v(C-C) gauche (1086) v_1_CO_3_^2−^,v_3_PO_4_^3−^,v(C-C) skeletal of acyl backbone in lipid (gauche conformation) (1087)
1125	Glucose (1117) C-C stretch (lipids) (1117–1119) C-O band of ribose (serves as a marker band for RNA in solutions) (1120) C-C stretching mode of lipids and protein, C-N stretch (1123–1130)
1162	C-C/C-N stretching (proteins) (1155–1158) Tyrosine (1163–1172) C-H in-plane bending mode of tyrosine (1170)
1250	Guanine, cytosine (NH2) (1250–1252) C-O_4_ aromatic stretch (1252) C-N in-plane stretching (1254) Lipids (1255) A, T (ring breathing modes of the DNA/RNA bases)-amide III (protein) (1257) Amide III, adenine, cytosine (1258) Guanine, cytosine (NH2) (1259) Amide II (1259)
1317	CH_3_/CH_2_ twisting, wagging &/or bending mode of proteins & lipids (1300–1314) G (ring breathing modes of the DNA/RNA bases)-C-H deformation (protein) (1318) Amide III (a-helix) (1318–1321)
1367	Tryptophan (1359–1369) v_s_(CH_3_) (phospholipids) (1367)
1428	v(C=O)O^−^ (amino acids aspartic & glutamic acid) (1400–1430) CH_2_ bending and scissoring modes of proteins & lipids, CH deformation (DNA/RNA & proteins & lipids & carbohydrates) (1420–1480) NH in-plane deformation (1423)
1495	Amide II (largely due to a coupling of CN stretching & in-plane bending of the N-H group, is not often used for structural studies per se because it is less sensitive and is subject to interference from absorption bounds of amino acid side chain vibrations) (1480–1575) DNA (1490) C-N stretching vibration coupled with the in-plane C-H bending in amino radical cations (1491)
1517	Cytosine (1506–1515) v(C-C), v(C=C) (1514–1538)
1542	Amide carbonyl group vibrations and aromatic hydrogens (1540–1680) Amide II (1544) C_6_-H deformation mode (1545) Bound & free NADH (1546) v(C=C), tryptophan (protein assignment) (1548–1560) v(CN) and d(NH) amide II (protein assignment) (1558) Tyrosine, amide II, COO^−^ (1558)
1567	Tryptophan (1560) Guanine, adenine, TRP (protein) (1573) Ring breathing modes in the DNA bases (1575) G, A (ring breathing modes of the DNA/RNA bases) (1575) C-C stretching (1580) d(C=C), C=C bending mode of phenylalanine (1582–1583)
1631	Tryptophan &/or b-sheet (1618–22) C¤=C¤ stretch (1628) Amide C=O stretching absorption for the ß -form polypeptide films (1628) Amide I band (both a-helix and b-structure) (1634–1645) Random coils (1647)

**Table 4:** Comparison between the different SERS spectra obtained. The band assignments that are common to the three spectra are indicated [11].

MBP	CSF	MBP + CSF	Proposed Band Assignment
848	839	858	Tyrosine (Fermi resonance of ring fundamental and overtone) (850–859) C-C stretch of proline ring, hydroxyproline, tyrosine ring breathing mode (852–856) (C-C) vibration of the protein backbone (856)
966	956	959	Single-bond stretching vibrations for the amino acids proline and valine (950) v_s_(CH_3_) of proteins (a-helix) (951)
1073	1081	1084	C-N stretching mode of proteins (1083)
1132	1120	1125	C-C stretching mode of proteins, C-N stretch (1123–1130)
1170	1164	1162	C-C/C-N stretching (proteins) (1155–1158) Tyrosine (1163–1172) C-H in-plane bending mode of tyrosine (1170)
1368	1367	1367	Tryptophan (1359–1369)
1475	1484	1495	Amide II (mainly due to a coupling of CN stretching & in-plane bending of the N-H group (1480–575) C-N stretching vibration coupled with the in-plane C-H bending in amino radical cations (1491)
1572	1573	1567	Tryptophan (1560) C-C stretching (1580) d(C=C), C=C bending mode of phenylalanine (1582–1583)
1609	1626	1631	Tryptophan &/or b-sheet (1618–1622) C¤=C¤ stretch (1628) Amide C=O stretching absorption for the ß -form polypeptide films (1628) Amide I band (both a-helix and b-structure) (1634–1645) Random coils (1647)

## Data Availability

Data are contained within the article.

## Supplementary Materials

The following supporting information can be downloaded at https://www.mdpi.com/article/10.3390/nano16100594/s1, Table S1: Normal results from the physicochemical analysis of CSF used in this study. Gram and bacteriological culture were negative; Figure S1: AFM height images of AgNPs; Figure S2: Absorbance spectroscopy UV-Vis showed a peak at 423 nm. Figure S3: Enhancement Factor calculation. References [18,19,20] are cited in the Supplementary Materials.
