# Supplementary material for: Raman and SERS Spectra of Human Myelin Basic Protein in Cerebrospinal Fluid
Source: Nanomaterials (Basel). 2026 May 12;16(10):594. doi: 10.3390/nano16100594 (PMC13209463; doi:10.3390/nano16100594)
Supplement: Supplementary file 1 [file nanomaterials-16-00594-s001.zip › nanomaterials-4308461-supplementary.pdf]

I. CSF reported analytics

Table S1. Normal results from the physicochemical analysis of CSF used in this study. Gram and bacteriological culture were negative.

Test	Value	Units	Test	Value	Units
Amount Received	1.0	mL	Appearance	TRANSPARENT	
Proteins	34.0	mg/dL	Glucose	49.0	mg/dL
Reference Value	12 - 60	mg/dL	Reference Value	40 - 70	mg/dL
Chloride	129.0	mEq/L	India Ink	NEGATIVE	
Cells per mm^3	2	c/mm^3	Red blood cells	2	c/mm^3
Leukocytes	0	c/mm^3	Differential count PMN	---	%
Differential count MN	---	%	Pandy test	NEGATIVE	

II. AgNP Characterization

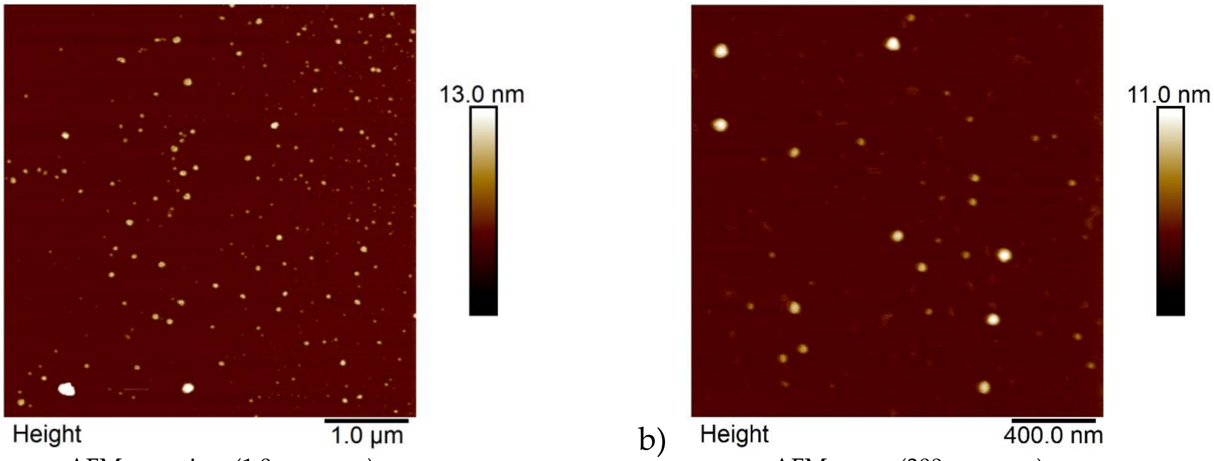

a) AFM overview (1.0 µm scan).

b) AFM zoom (300 nm scan).

Fig. S1. AFM height images of AgNPs. The overview scan shows particle distribution, and the higher-magnification image reveals individual nanoparticles and small clusters.

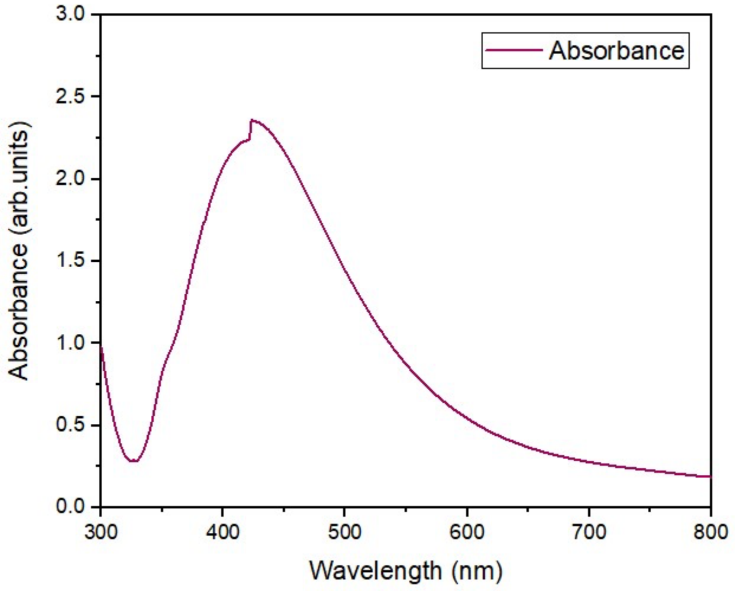

Fig. S2. Absorbance spectroscopy UV-Vis showed a peak at 423 nm.

III. Detailed Solution preparation.

Initially, we prepared MBP serial dilutions by mixing the purified protein with deionized water, obtaining the solutions with the following protein concentrations: C1=70 mg/ml, C3=0.7 mg/ml, C5=0.007 mg/ml, C7=70ng/ml, C9=0.7 ng/ml, C11=0.035 ng/ml

Spike-in experiments

a) Preparation for Raman: MBP+CSF

1) Control: 40 microliters of CSF + 10 µl deionized water
2) 40 µl CSF + 10 µl MBP C3
3) 40 µl CSF + 10 µl MBP C5
4) 40 µl CSF + 10 µl MBP C7
5) 40 µl CSF + 10 µl MBP C9
6) 40 µl CSF + 10 µl MBP C11

b) Preparation for SERS: MBP+CSF+AgNP

1) Control: 20 µl CSF + 5 µl deionized water + 25 µl AgNP
2) 20 µl CSF + 5 µl MBP C3 + 25 µl AgNP
3) 20 µl CSF + 5 µl MBP C5 + 25 µl AgNP

4) 20 µl CSF + 5 µl MBP C7 + 25 µl AgNP
5) 20 µl CSF + 5 µl MBP C9 + 25 µl AgNP
6) 20 µl CSF + 5 µl MBP C11 + 25 µl AgNP

To obtain the Raman and SERS spectra, 50 µl of each preparation was placed in wells on an aluminum plate, and all measurements were performed as described in the Methods section.

IV. Enhancement Factor calculation

The enhancement factor (EF) was calculated using the standard analytical SERS definition [S1]:

$$EF = \frac{I_{SERS}}{I_{Normal}} \frac{N_{Normal}}{N_{SERS}}$$

where I_{SERS} and I_{Normal} are the matched peak intensities (after baseline correction) under SERS and normal Raman conditions, respectively, and N_{SERS} and N_{Normal} are the estimated numbers of molecules contributing to each signal.
In the implemented physical model [S2]:

$$N_{Normal} = \frac{\pi r^2\, t(\rho \cdot 10^6)N_A}{M} \text{ and } N_{SERS} = \frac{\pi r^2}{\frac{A_{mol}}{10^{18}}}$$

With:

$$t = \frac{2\lambda}{(NA_{obj})^2} \text{ and } r = \frac{0.61\lambda}{NA_{obj}}$$

And $\lambda = 532\ nm$ the laser wavelength, $NA_{obj} = 0.4$ the numerical aperture of the objective, $N_A = 6.022 \cdot 10^{23} mol^{-1}$ the Avogadro's number, $A_{mol} = 35.5\ nm^2$the estimated effective superficial area of one molecule of the MBP, $\rho = 1.37\ \frac{g}{cm^3}$ the MBP's density, and $M = 16350\ \frac{g}{mol}$ the MBP's molar mass.
The MBP's molar mass was calculated as an average of the molar masses of isoforms (14.2 kDa and 18.5 kDa) present in the sample; the values were obtained from the UniProt database [S3]. For volumetric estimates, the protein density was assumed according to the experimental value reported by Fischer et al. [S4]. From these values, the molecular volume was calculated, and, assuming a spherical geometry, the effective surface area was estimated; this calculation provides an approximate ideal value because it does not consider the protein´s intrinsically disordered nature.

The enhancement factor was computed for the three most intense peaks, as shown in Figure S3.

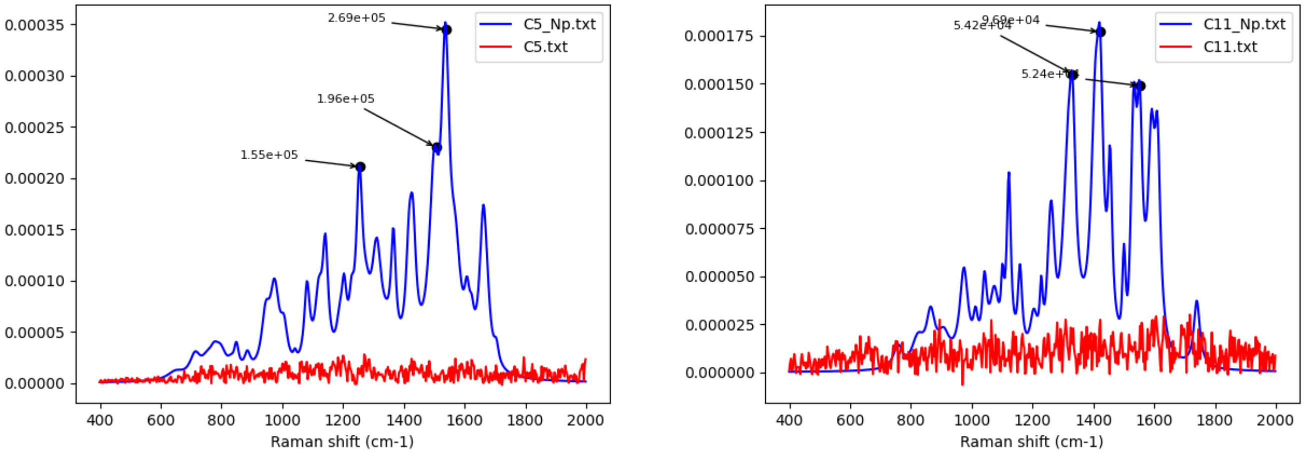

Fig. S3. Enhancement Factor calculation. In this Figure, the Raman spectrum is shown in red and the SERS spectrum in blue; both spectra were obtained on the same aluminum plate.

[S1] Le Ru, E.C.; Blackie, E.; Meyer, M.; Etchegoin, P.G. Surface enhanced Raman scattering enhancement factors: A comprehensive study. J. Phys. Chem. C 2007, 111(37), 13794-13803. https://doi.org/10.1021/jp0687908

[S2] Neupokoeva A.; Bratchenko I.; Bratchenko L.; Khivintseva E.; Shirolapov I.; Shusharina N.; Khoimov M.; Zakharov V.; Zakharov A. Raman liquid biopsy: a new approach to the multiple sclerosis diagnostics. Front Neurol. 2025, 16, 1516712. https://doi.org/10.3389/fneur.2025.1516712

[S3] *UniProt*. (n.d.). UniProt. https://www.uniprot.org/uniprotkb/P02686/entry

[S4] Fischer, H., Polikarpov, I., & Craievich, A. F. (2004). Average protein density is a molecular-weight-dependent function. *Protein Science*, *13*(10), 2825–2828. https://doi.org/10.1110/ps.04688204
